# Intrahepatic Cholestasis of Pregnancy: A Case Study of the Rare Onset in the First Trimester

**DOI:** 10.3390/medicina55080454

**Published:** 2019-08-09

**Authors:** Milos Stulic, Djordje Culafic, Ivan Boricic, Milica Stojkovic Lalosevic, Nina Pejic, Goran Jankovic, Tamara Milovanovic, Violeta Culafic-Vojinovic, Zeljko Vlaisavljevic, Milica Culafic

**Affiliations:** 1 Clinic for Gastroenterology and Hepatology, Clinical Center of Serbia, School of Medicine, University of Belgrade, 11000 Belgrade, Serbia; 2 Institute of Pathology, School of Medicine, University of Belgrade, 11000 Belgrade, Serbia; 3 Department of Internal Medicine, General Hospital Euromedic, 11000 Belgrade, Serbia; 4 Department of Pharmacokinetics and Clinical Pharmacy, Faculty of Pharmacy, University of Belgrade, 11000 Belgrade, Serbia

**Keywords:** intrahepatic cholestasis of pregnancy, the first trimester, total serum bile acids, pruritus

## Abstract

Intrahepatic cholestasis of pregnancy (ICP) is a gestation-specific liver disorder, defined most often as the onset of pruritus, usually from the third trimester of pregnancy, associated with abnormal liver test results and/or increased total serum bile acids and spontaneous relief after delivery. The 21-year-old patient was admitted to our ward in the 11th week of pregnancy due to raised liver enzymes. The first onset of pruritus and jaundice appeared a month before hospitalization. Immunology tests and *Toxoplasma gondii* were negative. We excluded viral etiology, while alpha-1-antitrypsin, serum and urine copper levels, and thyroid hormones were within the reference values. The patient denied she had taken any medicines and herbal preparations before and during pregnancy. Total bile acids in the serum were significantly elevated (242 μmol/L). The abdominal ultrasound revealed a regular finding. Liver biopsy suggested a cholestatic liver disorder. After a presentation of all risks, the patient decided to stop the pregnancy. After a month, the hepatogram was within the reference values. Very rarely an ICP can occur in early pregnancy (first trimester), which calls for close monitoring. The risk of serious adverse fetal outcomes and spontaneous preterm delivery is proportional with increased levels of maternal serum bile acid.

## 1. Introduction

Intrahepatic cholestasis of pregnancy (ICP) was firstly described in 1883 [1]. It is a gestation-specific liver disorder, defined most often as onset of pruritus, usually in the third trimester of pregnancy. The main indicators are elevated liver enzymes and/or increased level of total serum bile acids (TSBA), and spontaneous resolution following delivery, in the absence of other skin or liver diseases [2]. ICP is the first cause of jaundice in pregnancy after exclusion of viral hepatitis [3]. The prevalence of this liver disorder varies according to geographical location and ethnicity, as genetic and environmental factors are recognized to be a part of its manifestation [4]. The range of ICP has been calculated between 0.01% and 0.2% in North America and Southern Europe, 0.8%–1.5% in South Asia [5], 0.6% in South Australia [6], between 1.5% and 4.0% in South America [7]; and 1.5% in Scandinavia [2]. The frequency of recurrence in future pregnancies has been noted to be between 40% and 60% and severity varies in the following pregnancies in a haphazard manner [8]. Among the most affected countries in the world are Chile, Bolivia, Finland, Sweden, and Portugal [4].

Severe ICP (defined by most authors as when TSBA is greater than 40 μmol/L) [2] appears to be linked to an increased proportion of serious adverse fetal outcomes including fetal distress, sudden intrauterine death (possibly due to an acute anoxic event [9], or impaired fetal cardiomyocyte function [10], preterm labor, meconium staining of amniotic fluid (MSAF), low birth weight, or respiratory distress syndrome of the neonates [2,3,4,5,6,7,8,9,10,11].

Gynecologists decrease the complication rates, including perinatal death, by delivery at the 36th week of gestation [12]. Maternal prognosis is usually satisfactory. Except severe pruritus, the main problem could be a higher risk of postpartum bleeding [13]. The reason is that ICP causes impaired absorption of fat-soluble vitamins (D, E, A, K) from the terminal ileum, due to altered enterohepatic circulation of bile acids. As the most important vitamin for coagulation, vitamin K is synthesized by the mother, and the fetus takes it by transplacental passage [14].

In the study of Swedish women with births between 1973 and 2009, researchers identified 11,388 women with ICP who were matched to 113,893 women without this diagnosis. Later, hepatobiliary diseases were more often confirmed in women with ICP (hazard ratio (HR) 2.62), hepatitis C or chronic hepatitis (HR 4.16 and 5.96, respectively), fibrosis/cirrhosis (HR 5.11), gallstone disease or cholangitis (HR 2.72 and 4.22, respectively) as compared to women without ICP (*p* < 0.001 for all HRs) [15].

## 2. Case Report

**Figure 1:**
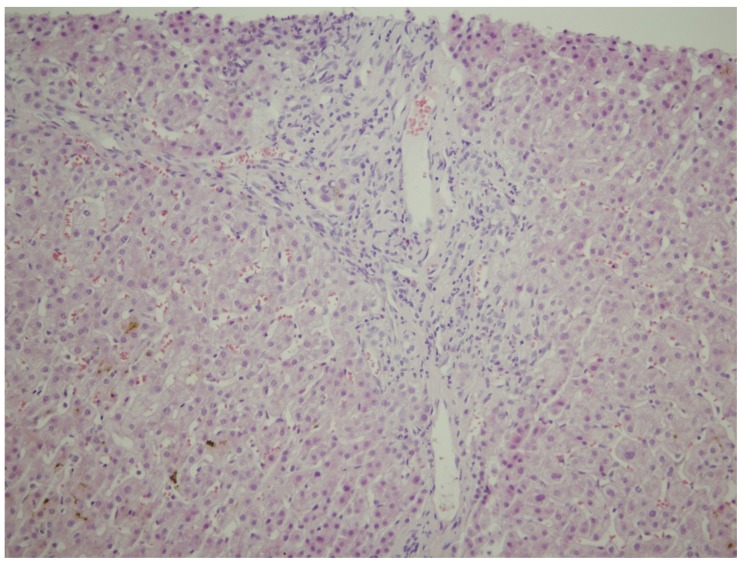
Mild, predominately lymphocytic inflammatory infiltrate of portal tract, admixed with some neutrophils (HEx200).

**Figure 2:**
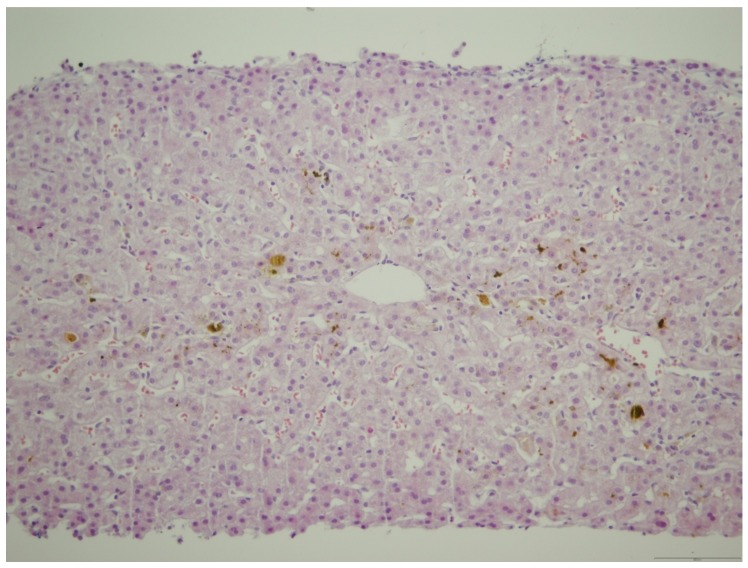
Moderate canalicular and mild cellular cholestasis with mild inflammatory reaction in the sinusoids (HEx200).

The 21-year-old patient was admitted to our ward in the 11th week of the first spontaneous pregnancy due to raised liver enzymes. In a personal history, she denied other diseases, operations, and allergies. After the third week of pregnancy, during routine screening, mild elevation of transaminases was noted. In the ninth week of pregnancy, the patient presented with pruritus and jaundice, which persisted for two weeks. The itching was most pronounced on the palms and soles of the feet. The laboratory screening revealed the following: Aspartate aminotransferase (AST) 246 IU/L, alanine aminotransferase (ALT) 476 IU/L, alkaline phosphatase (ALP) 120 IU/L, gamma-glutamyltransferase (GGT) 55 IU/L, total bilirubin 72 μmol/L, direct bilirubin 46 μmol/L. Other measured parameters were in a reference range (blood count, electrolyte status, renal, and synthetic liver function). Viral etiology was also negative (HBsAg, HCV, HAV, HEV, HIV, CMV, EBV, HSV 1, HSV 2, Adeno, Parvo B19, Coxsackie). *Toxoplasma gondii* was negative. Furthermore, thyroid hormone values were within the reference values. The patient denied she had taken any medicines and herbal preparations before and during pregnancy. Immunological analysis (ANA, ASMA, AMA, ANCA, and LKM1) were negative, alpha-1-antitrypsin, serum, and urine copper levels were within the reference values. However, the concentration of TSBA was significantly elevated (242 μmol/L, reference value up to 10 μmol/L). We verified the deterioration of the hepatogram: AST 422 IU/L, ALT 759 IU/L, ALP 150 IU/L, GGT 115 IU/L, total bilirubin 114 μmol/L, direct bilirubin 91 μmol/L. Abdominal ultrasound verified an average-size liver without gallstones; extrahepatic or intrahepatic bile ducts were not dilated, and there were no signs of portal hypertension or other vascular abnormalities. Continual gynecological observation confirmed the normal development of the fetus. We performed a liver biopsy with a Menghini needle, with a diameter of 1.4 mm, and pathohistological finding suggested a cholestatic liver disorder (Figure 1 and Figure 2).

We started treatment with ursodeoxycholic acid (UDCA), 1 g daily (10–20 mg/kg), divided into three doses. After a consultative session (hepatologist, gynecologist, and anesthesiologist) and presentation of all the risks, the patient decided to terminate the pregnancy. The Collegium of the Clinic for Gastroenterology and Hepatology and the Collegium of the Clinic for Gynecology and Obstetrics, Clinical Center of Serbia, approved that the intervention could be performed with the written informed consent of the patient. After that, the termination of pregnancy was done without complications. The hepatogram returned to normal a month after the procedure.

## 3. Discussion

Most often the disease affects women in the third trimester of pregnancy with a history of intrahepatic cholestasis during previous pregnancies [7], history of cholestasis related to the use of oral contraceptives [16], family or personal history of biliary disease [17], hepatitis C viral infection [18], twin pregnancies [19], or in vitro fertilization pregnancies [20]. It is also implied that the risk of acquiring ICP is greater in women over the age of 35 [21].

Our patient is a rare example of the onset of ICP in the first trimester of pregnancy without any of the above risk factors. A comprehensive review of the literature showed only a few cases of severe, first-trimester ICP.

Hubschmann et al. reported a case study of a 26-year-old pregnant Guatemalan female with diffuse generalized itching, dark urine, and light-colored stools at the seventh gestation week. The patient reported a history of intense pruritus with elevated transaminases and bilirubin while using some estrogen/progestin combination hormonal contraceptive pills. Furthermore, she had two previous pregnancies in which ICP was manifested in the 20th and 10th gestational week, respectively. The second one resulted in stillbirth at the 27th week of pregnancy. This time, the patient had moderate elevation of the liver enzymes, while TSBA at the 13th gestational week was 243.6 µmol/L. Compared to our patient, this is the only case where a higher TSBA level during the first trimester of pregnancy has been described. Liver biopsy was performed at the 22nd week of pregnancy and histology revealed pure intrahepatic cholestasis. The patient received treatment (UDCA, S-adenosyl methionine, and cholestyramine) and with close monitoring, she delivered a viable female infant weighing 1758 g [22].

Keitel et al. presented a case of ICP in the first trimester of pregnancy, in a 26-year-old woman, with extremely high transaminase levels. The peak increase of ALT level was 1704 IU/L at the 13th gestational week, while concentration of TSBA was 98 µmol/L. She stated that pruritus was also induced three years earlier by the contraceptive pills. Her mother and aunt had a history of the third trimester pruritus and premature birth. Genetic analysis indicated ICP. In this case also, the authors decided to perform a liver biopsy at the 11th week of pregnancy. Histologic analysis showed mild cholestasis, minimal portal inflammation, and single cell necrosis. The patient was treated with UDCA, and, in the 35th week of pregnancy, she had a preterm rupture of membranes, followed by a spontaneous delivery of a healthy female infant [23].

The etiology of ICP is multifactorial and involves hormonal, genetic, and environmental factors. Multiple pregnancies have five times greater prevalence of ICP because of higher levels of estrogens in comparison with singleton pregnancies [24]. The association between high levels of estrogen and intrahepatic cholestasis has been demonstrated also in genetically predisposed persons with reduced sulfation and impaired transport of bile acids. The usage of oral contraceptives with high estrogen content can lead to a cholestatic situation that is similar to ICP [25]. Unlike for estrogen, the connection between progesterone and ICP is very unclear. In a study by Reyes et al. [26], it has been suggested that genetic polymorphism of canalicular transporters for steroid sulfates or their regulation in patients with ICP could be a reason of a selective defect in the secretion of sulfated progesterone metabolites into bile.

An ICP-associated gene has been described to be located in the p23 region of chromosome 2 [27]. Changes in the structure of cell membranes of bile ducts and hepatocytes, as well as in following dysfunction of biliary canalicular transporters, may be due to genetic predisposition [28]. Several studies reported mutations in the hepatic phospholipid transporter (MDR3/ABCB4) and aminophospholipid transporter (ATP8B1/FIC1) in patients with ICP [24,25,29]. Moreover, it is important to emphasize that bile salt secretion from hepatocytes into bile in humans is done through the bile salt export pump (BSEP/ABCB11), which is the major transporter as a member of the ATP-binding cassette superfamily [30].

Floreani et al. [31] suggested that the gamma-aminobutyric acid (GABA) system has a very important role in the pathophysiology of ICP based on finding gamma-aminobutyric acid type A receptor alpha2 subunit (*GABRA2*) gene upregulation in those cases.

In recent years, it has been confirmed that there is a correlation between obstetric complications with the presence of fetal deoxyribonucleic acid (DNA) in the maternal plasma. Yi et al. suggested that raised circulating hypermethylated RAS-association domain family 1, isoform A (*RASSF1A*) gene sequences may serve as a diagnostic marker for ICP [32].

Geographic and seasonal conditions are environmental factors that can lead to the expression of ICP in women with genetic predisposition [25]. A higher number of cases in January may insinuate a higher incidence of ICP in winter [17,18,19,20,21,22,23,24,25]. Variations in diet that involve the ingestion of food with high levels of copper and low levels of selenium and zinc may be associated with seasonal differences in the incidence of the disease. Possibly these variations led to the decreased prevalence of ICP in Chile from a range of 11.8%–27.7% during the 1970s (the higher value observed for Araucanian ethnicity) [33] to the range of 1.5%–4.0% in the 1990s [7].

Selenium has been described as a co-factor of a number of enzymes in oxidative metabolism in the liver, but its role in bile secretion remains unclear. However, there is some evidence that long-chain monounsaturated fatty acids and low selenium levels could be related to etiopathogenesis of ICP [4,26,34].

Estiu et al. reported a study of 382 pregnant women with ICP. Only six cases were diagnosed before the 24th gestational week, and two of them had a severe form with TSBA greater than 40 μmol/L. Authors presented the meconium risk factor (MRF)—defined as the ratio between serum bile acid concentrations and the gestational age in weeks, both at the time of diagnosis. The probability of MSAF was enhanced four-fold in ICP patients with MRF > 3. A statistically significant correlation was established between higher serum levels of ALT, ALP, and direct bilirubin with increased frequency of MSAF [35].

The MRF of our patient was 22, while ALT showed a 19-fold increase and direct bilirubin a 14-fold increased, hence the risk of MSAF was extremely high.

Moreover, researchers reported a stillbirth incidence of 1.4% (10 cases out of 719 women with severe ICP). The risk was greater in seven of 10 ICP cases with coexisting complications. There were two cases with preeclampsia, three with gestational diabetes, and two with nonspecified complications. Further analysis confirmed that a double increase in the level of TSBA increased the risk of all preterm delivery by 68%, spontaneous preterm delivery by 66%, MSAF by 55%, and stillbirth by 200% [36].

ICP is associated with elevated aminotransferases, especially ALT, which can be 2–10-fold increased. Alkaline phosphatase is commonly elevated in pregnancy, while GGT could be mildly increased, but it is usually normal [4]. Our patient had a significant elevation of transaminases, while ALT was 20-fold increased. Since she was in the first trimester of pregnancy, we performed all available serological tests to exclude many hepatological diseases (infectious, autoimmune, Wilson’s disease, etc.), while genetic testing was not available to us, a Collegium decision was to perform a liver biopsy. The patient was presented with the risks, and with her written informed consent, a liver biopsy was performed without any complications.

We also thought about the unusual manifestations of some very rare hepatological diseases. Alagille syndromes are usually manifested in infancy or early childhood, while pathohistological findings indicate reduction or atresia of the bile ducts [37] which was not the case in our patient.

Benign recurrent intrahepatic cholestasis (BRIC) type 1 and 2 are acute cholestatic disorders of adolescence and adulthood. It is characterized by acute episodes of cholestasis, jaundice, and severe pruritus caused by unknown factors, which after weeks or months completely resolve to start again after an asymptomatic period of months to years. The cholestatic episodes generally begins before the second decade of life while pathohistological findings usually indicate liver fibrosis [38].

It has been found that TSBA levels were higher in vitro fertilization (IVF) than in the spontaneous one. The elevated TSBA levels in the IVF pregnancies might be elucidated by higher rates of multiple pregnancies and hormonal therapy. However, no difference between IVF and natural fertilization in term of age, onset time of symptoms, ALT, alkaline phosphatase, total and direct bilirubin levels, prothrombin time, international normalized ratio, and platelet count were observed [39].

A recent meta-analysis has shown that timely introduction of UDCA and close monitoring of ICP patients are beneficial and lead to a reduction in the rate of adverse pregnancy outcomes. In the long term, the ICP could affect health of the offspring, including sensibility to increased adiposity and metabolic disease [40]. The dose of 10–20 mg/kg of UDCA per day is still regarded as the first-line treatment for ICP based on evidence obtained from randomized clinical trials. Serum liver tests could be improved in 67%–80% of ICP patients treated with UDCA. Nevertheless, the reduction of fetal complication rate is vague as fetal complication rates were low in recent trials both in UDCA and placebo-treated patients. S-Adenosyl-l-methionine is less effective than UDCA but may have an additive effect [41,42].

Dexamethasone (12 mg/day for seven days) promotes fetal lung maturity. However, it is ineffective in decreasing ALT levels and pruritus in ICP patients [42,43]. A common practice is the induction of labor at the 36th–38th week of gestation to lower the risk of intrauterine death [2,3,4,5,6,7,8,9,10,11,12,13,14,15,16,17,18,19].

Rifampicin enhances bile acid detoxification, an effect that is complementary to the upregulation of bile acid export induced by UDCA, suggesting that the two drugs used in combination may be more effective than monotherapy [44]. Geenes et al. reported a study of 27 pregnant women with ICP. They were using UDCA as monotherapy, but TSBA remained high. In 14 pregnancies, TSBA decreased following the introduction of rifampicin. Authors suggested that combined treatment with UDCA and rifampicin is an effective way of treating women with severe ICP who do not respond to treatment with UDCA alone [45].

## 4. Conclusions

Very rarely an ICP can occur in early pregnancy (first trimester), which calls for close monitoring. The risk of serious adverse fetal outcomes and spontaneous preterm delivery is proportional with increased levels of maternal serum bile acid.
